# What Caused Seriously Shortage of Chinese Nurses?

**Published:** 2018-07

**Authors:** Min ZHOU, Lindu ZHAO, Nan KONG, Kathryn S CAMPY, Shujuan QU

**Affiliations:** 1. College of Business Administration, Hunan University of Commerce, Changsha, China; 2. School of Economics and Management, Southeast University, Nanjing, China; 3. Weldon School of Biomedical Engineering, Purdue University, West Lafayette, USA; 4. Center for Public Health Initiatives, University of Pennsylvania, Philadelphia, PA, USA; 5. The Third Xiangya Hospital of Central South University, Changsha, China

## Dear Editor-in-Chief

Since 2009, China has implemented the “New Medical Reform” and has achieved remarkable success: China has built the largest basic medical security networks in the world which covered 98% populations and benefiting 1.3 billion people in 2017; more than 80% of residents can reach the nearest medical center within 15 minutes; the average life expectancy of Chinese people was exceeds 76.3 years. Undoubtedly, China’s health care workers play an important role in the “New Medical Reform” (1).

By the end of 2017, China had 8.91 million health technicians, including 3.35 million physicians and 3.79 million registered nurses. In addition, China has built 30000 hospitals, 94000 township clinics and 7.85 million beds. In 2017, Chinese medical institutions completed 8.10 billion medical visits and 240 million inpatients. In recent years, the nurse shortage in China has become more and more acute (2).

According to National Bureau of Statistics of China in 2018 and the World Bank in 2017, the number of nurses in China is the largest in the world. However, China also holds the largest population. The number of nurses per 1,000 people is only 2.73, which is far below the average of the developed countries: United States (2014) 8.76, Japan (2015) 11.46, Germany (2015) 13.51. As to the ratio of nurses/physicians, the situation is even more severe: China (2017) 1.13, United States (2014) 3.31, Japan (2015) 5.00, Germany (2015) 3.30, India (2016) 2.80 (Table 1).

**Table 1:** Comparison of nurse resources in China and major countries in the world

***Indicators***	***China (2017)***	***United States (2014)***	***Japan (2015)***	***Germany (2015)***	***India (2016)***
Population (Million)	1390.08	321.78	126.58	80.69	1324.17
Number of beds (Million)	7.85	0.94	1.73	0.66	0.93
Number of physicians (Million)	3.35	0.85	0.29	0.33	0.94
Number of nurses and midwives (Million)	3.79	2.82	1.45	1.09	2.63
Number of beds per thousand	5.65	2.91	13.70	8.20	0.70
Number of physicians per thousand	2.41	2.65	2.29	4.09	0.71
Number of nurses per thousand	2.73	8.76	11.46	13.51	1.99
Nurse physician ratio	1.13	3.31	5.00	3.30	2.80

Source: National Bureau of Statistics of China (www.stats.gov.cn); World Bank Database (https://datacatalog.worldbank.org)

China faces two major challenges of childbirth peaks and population aging, both of which will greatly increase the demand for nurses. From January 1, 2016, China officially implemented the “two-child” policy, replacing the policy of “one child for a couple” which have implemented nearly 40 years. Since the implementation this policy two years, the population of newborns in China has increased significantly (3). In 2016 and 2017, the birth population of China is 17.86 million and 17.23 million, which was 1.42 million and 790,000 more than the annual average number of births before the implementation of the policy. In 2017, there were only 0.15 pediatric nurses per 1,000 children aged 0–14 in China. On the other hand, the elderly in China has rapidly increased to 241 million people (2017) and is expected to increase to 248 million by 2020. Depending on the recommendations from WHO, the number of nurses per 1,000 people in an aged society must not less than eight (4). To meet this standard, China is required to train qualified nurses and midwives 7.33 million in three years. Obviously, it is extremely difficult to accomplish.

While the demand for nurses is growing rapidly, the number of admissions to nursing colleges, the employment intention of nursing graduates, and the attrition rate of nurses who are already employed are very worrying. According to the data released by the Ministry of Education of China, the enrollment scale of nursing undergraduates is approximately 44,000 (2016), the enrollment scale of vocational schools is approximately 31,000 (2016), and the employment rate is approximately 85%. In terms of employment intentions, 65.8% of nursing graduates are engaged in clinical nursing work, 23.1% of respondents are engaged in medical-related work such as basic medical research, medical management and drug sales, and 11.1% of the respondents are Not in the medical industry. In addition, according to statistics from the National Health and Family Planning Commission, the average turnover rate of nurses is 8.2%, and 12.6% of departing employees will no longer work as a nurse. Reasons for resignation mainly include: regular overwork (24.5%), high work pressure (17.1%), tense nurse-patient relationship (15.3%), and low salary (12.8%). According to the above data analysis, there will be no significant increase in the number of nurses in China. Because Chinese nursing staffs have been in a high-intensity work environment for a long time, 75.4% of nurses have clearly stated that they are tired of nursing work.

The rapid growth in nursing demand and the slow increase in the number of qualified nurses has led to a serious shortage of nurses in China. With the accelerated growth of the aging population and newborns, this problem will become even more desperately. Because the changes in China’s demographic structure have been unavoidable, China’s wisest choice is to reduce the workload and work pressure of nurses and significantly increase nurses’ remuneration. The government should take measures to attract more students work as a nurse, and by this way to alleviate the upcoming grim reality.
